# Breeding and Disease Resistance Evaluation of a New Largemouth Bass (*Micropterus salmoides*) Strain Resistant to Largemouth Bass Virus (LMBV)

**DOI:** 10.3390/ani15243510

**Published:** 2025-12-05

**Authors:** Wenxian Li, Pinhong Li, Xia Luo, Ningqiu Li, Qiang Lin, Hongru Liang, Yinjie Niu, Baofu Ma, Wenwen Xiao, Xiaozhe Fu

**Affiliations:** 1 Pearl River Fishery Research Institute, Chinese Academy of Fishery Sciences, Key Laboratory of Fishery Drug Development, Ministry of Agriculture and Rural Affairs, Guangdong Province Key Laboratory of Aquatic Animal Immune and Sustainable Aquaculture, Guangzhou 510380, Chinalxwenhao@163.com (X.L.); lin9902057@163.com (Q.L.); mabf@prfri.ac.cn (B.M.);; 2 College of Fisheries and Life Science, Shanghai Ocean University, Shanghai 201306, China

**Keywords:** largemouth bass, LMBV, disease resistance, immune-related gene, antioxidant capacity

## Abstract

Largemouth bass virus (LMBV) has caused severe impacts on the largemouth bass aquaculture industry in China. Breeding new virus-resistant strains is a fundamental strategy to address the LMBV challenge. In this study, a candidate LMBV-resistant population of largemouth bass was developed using the “Youlu No. 3” as the base population. Through three consecutive generations of selective breeding, a new LMBV-resistant strain was successfully established. To evaluate the LMBV resistance of different breeding generations, four key indicators, including survival rate post-LMBV infection, LMBV viral load, the expression of immune-related genes (GADD45b, FOXO3, TNF-α, IFN-γ, and IL-10), and antioxidant enzyme activities (GSH and AKP), were analyzed in this study. Notably, the F3 generation exhibited significantly lower viral loads in liver tissues after LMBV infection compared to the F1, F2, and control groups. Furthermore, the F3 generation showed a markedly higher survival rate at 14 days post-challenge, indicating enhanced disease resistance. Additionally, both the F2 and F3 generations had increased expression levels of the immune-related genes and elevated activities of antioxidant-related enzymes. These results collectively indicate that the F3 generation possesses stronger LMBV resistance than the F2, F1, and control groups. This study provides effective strategy for addressing LMBV disease in largemouth bass at the source and is of great significance for promoting the healthy and sustainable development of the largemouth bass aquaculture industry.

## Simple Summary

Largemouth bass virus (LMBV) has inflicted significant losses on the largemouth bass aquaculture industry, necessitating the urgent development of virus-resistant strains. This study utilized “Youlu No. 3” as the foundational population and performed three successive generations of selective breeding (F0 to F3) to cultivate largemouth bass resistant to LMBV. Notably, all test fish across generations preserved a 1:1 male-to-female ratio. In study, we assessed four critical parameters: survival rate post-LMBV infection, viral load, expression of immune-related genes, and activities of antioxidant enzymes. The findings indicated that the F3 generation exhibited superior performance, with a final survival rate of 52.5%, markedly surpassing that of the F1 (10%), F2 (25%), and control group (5%), alongside a much-reduced viral burden. The F3 generation exhibited improved immune gene expression and elevated antioxidant enzyme activity, although its growth performance remained consistent with earlier generations. This research successfully established a novel LMBV-resistant strain of largemouth bass (F3), offering an effective strategy to mitigate LMBV outbreaks and promoting the healthy and sustainable advancement of the largemouth bass aquaculture industry.

## 1. Introduction

The largemouth bass (*Micropterus salmoides*), also known as the California bass, is native to the Mississippi River basin in North America. Largemouth bass are highly regarded by consumers for their fast growth, low intramuscular bones, and delicious meat, among other characteristics [1]. The total output of largemouth bass in China reached 888,000 tons in 2023, representing a year-on-year increase of 10.66%, with aquaculture production steadily rising each year [2]. However, the intensive aquaculture of largemouth bass has made it increasingly susceptible to disease outbreaks, including parasitic diseases, bacterial diseases, and viral diseases [3]. Notably, LMBV has been identified as a cause of some of these cases. LMBV, a member of the genus Ranavirus (family *Iridoviridae*), was initially prevalent in wild natural waters of largemouth bass [4]. LMBV, a highly pathogenic ranavirus, was first isolated in China from Guangdong Province in 2008 [5] and has since attracted significant public attention [6]. Notably, Asian strains of LMBV have demonstrated a broader pathogenicity range than those found in the United States [7]. Following its emergence, the virus spread throughout major largemouth bass farming regions in China, with numerous mass mortality events subsequently reported [8]. LMBV has been successfully isolated from more than 16 fish species, and various freshwater fish, including perch, carp, snakehead fish, and hybrid mandarin, were susceptible to LMBV and exhibited different pathogenicity levels and clinical symptoms [9]. Recent studies confirm that LMBV is highly lethal and infectious, capable of infecting fish of various sizes. The mortality rate of adult fish infected with LMBV ranges from 10% to 40% [10], which is potentially influenced by genetic background and variations in immune-related genes. LMBV exhibits a broad transmission range, facilitated by its optimal replication temperature of 24–28 °C. This range closely coincides with the preferred water temperatures for largemouth bass growth (25–30 °C) [11]. Experimental intraperitoneal injection in young fish induces typical acute symptoms, including hemorrhage and visceral enlargement, with mortality showing a clear dose-dependent relationship.

Selective breeding is a long-term selection process with significant potential for genetic improvement [12]. Family selection combined with molecular marker-assisted selection (MAS) can be used for precise targeting of desired traits in largemouth bass and significant enhancement of breeding efficiency. This strategy has been successfully applied in breeding programs for aquatic species such as mandarin fish (*Siniperca chuatsi*) [13], Atlantic salmon (*Salmo salar*) [14], and Australian blue mussel (*Mytilus galloprovincialis*) [15]. Sun et al. [16] detected significant differences in disease resistance among various largemouth bass lineages by evaluating their survival rates after LMBV infection, indicating that it is feasible to use family selection to improve resistance to LMBV. Compared with family selection alone, the combination of family selection and MAS can more effectively identify functional genes associated with disease resistance and reveal the regulatory mechanisms. Notably, in the comprehensive genome analysis of largemouth bass resistance to LMBV SNP sites, 1018 candidate disease resistance genes were identified, followed by KEGG enrichment analysis, which included MHC II, p38 MAPK, AMPK, SGK1, FOXO3, FOXO6, S1PR1, IL7R, RBL2, and GADD45b [17]. To reduce the impact of viral diseases on largemouth bass, researchers have initiated breeding programs focused on developing resistance to LMBV. By now, our team has successfully completed the selection and breeding of LMBV resistance from the F0 to F3 generations.

In the present study, we developed a candidate LMBV-resistant largemouth bass population by evaluating a comprehensive set of criteria, including survival rate after LMBV challenge, viral load, antioxidant enzyme (GSH, AKP) activities, and immune-related gene expression. Disease resistance was significantly enhanced over successive generations, with the F3 generation exhibiting notably robust resistance. These results indicated that a new LMBV-resistant strain was successfully established through three consecutive generations of selective breeding.

## 2. Materials and Methods

### 2.1. Fish and Virus

A total of 5100 Youlu No. 3 (average body length 4.0 ± 0.5 cm, average body weight 4.0 ± 1.0 g) were purchased from Foshan as broodstock. And antiviral screening tests were performed on these fish and utilized for breeding purposes. Firstly, ten experimental fish were randomly tested for viruses (Viruses were identified by quantifying the viral copy number in tissues), parasites (parasites were identified through microscopic analysis of gill filaments and fin rays obtained from the fish), and bacteria (bacteria were identified by isolating and growing them from tissue samples via plating). The qualified fish were then used to establish 50 half-sib families for subsequent breeding purposes. In the infection experiment, each tank housed 200 fish (as water quality becomes unmanageable when the fish count exceeds this limit), and the number of tanks utilized was based on the total number of experimental fish in each generation’s infection trial. This infection experiment could efficiently differentiate between two cohorts exhibiting strong and weak disease resistance: fish who perished during the viral onset were categorized as the virus-susceptible group, whilst the few surviving fish were designated as the virus-resistant group. For the susceptible group, internal organs and caudal fins were collected and immersed in 100% ethanol and preserved at −80 °C; for the resistant group, caudal fins were collected, immersed in 100% ethanol and preserved at −80 °C. All fish were maintained in tanks supplied with ample aeration in a flow-through system, fed daily with commercial feed, and reared at a water temperature of 28–30 °C.

LMBV isolates were isolated and stored in our laboratory [18]. These viruses were propagated in CPB cells cultured with L-15 medium supplemented with 2% FBS at 28 °C, and the viral supernatants were stored at −80 °C [19].

### 2.2. Generational Breeding of Largemouth Bass for Disease Resistance

The experimental fish were anesthetized with glycerol and injected intraperitoneally with 0.1 mL of LMBV solution at a concentration of 1 × 10^5^ TCID_50_/mL. During the experimental period, the dissolved oxygen content was maintained at >5 mg/L, and the water temperature was maintained at 28–30 °C. The fish were continuously observed for 10 days after the injection, and the daily survival rates of each group were recorded [20]. Dead fish were collected and stored according to standard protocols. The surviving fish were used as broodstock, designated as F0. The surviving parental families were monitored every 15 days for viral load to confirm complete clearance. Upon reaching gonadal maturity, the fish successfully spawned. The fertilized eggs were later hatched, and the fry were reared to the target experimental size (8.0 ± 0.5 cm, 8.0 ± 1.0 g). The subsequent generation of broodstock was then evaluated through further challenge experiments. Several families established via interline crossing served as the experimental population for assessing LMBV resistance.

### 2.3. Comparison of Survival Rates Across the Three Breeding Generations After LMBV Infection

The largemouth bass (F1, F2, F3, and the control group) were initially categorized into four respective experimental groups. To ensure the reliability of experimental results and reduce random errors, each of the four groups was assigned three parallel controls. Consistent with this categorization and parallel control design, the bass were allocated to 12 culture tanks (i.e., 4 groups × 3 parallel controls = 12 tanks), with 40 fish assigned to each tank to ensure uniform stocking density throughout all experimental units. Experimental fish were anesthetized with glycerol and injected intraperitoneally with 0.1 mL of LMBV solution at a concentration of 1 × 10^5.5^ TCID_50_/mL. During the experiment, observations were conducted every 2 h, and the dead fish were removed in a timely manner, and the survival rate of each group was calculated at the end of the experiment.

### 2.4. Viral Load Dynamics

To analyze viral load dynamics, largemouth bass were randomly divided into four groups (F1, F2, F3, and a control), with 35 fish per group held in three parallel tanks. The fish were injected intraperitoneally at a dose of 10^4.5^ TCID_50_/mL per fish. Liver tissues were collected at 0, 12, 24, 72, and 168 hpi, respectively. Viral load was quantified by quantitative PCR (qPCR) [20], targeting the LMBV major capsid protein (MCP) gene to compare dynamics among the groups. Total genomic DNA was extracted from the liver samples, and viral copy numbers were subsequently measured at 24 and 72 hpi.

### 2.5. Expression of Immune-Related Genes

#### 2.5.1. Challenge Experiment and Sample Collection

To examine the expression of immune-related genes, spleen tissues were collected from challenged largemouth bass (F1, F2, F3, and control groups) with three biological replicates (3 fish per replicate) per group at each time point, at 0, 12, 24, 72, and 168 hpi, as described in Section 2.4.

#### 2.5.2. RNA Extraction and Tr2anscription

Total RNAs were isolated from the spleen tissues taken from three fish from F1, F2, and F3 at 6 h, 12 h, 24 h, and 72 hpi with the RaPure Total RNA Kit (Magen, Guangzhou, China), and on-column DNase treatment was performed to remove genomic DNA contamination during the extraction process. After RNA isolation, the concentration of total RNA was measured using a Cytation 5 Cell Imaging Multimode Reader (BioTek, Winooski, VT, USA) to ensure the quality of RNA for subsequent experiments. The RNA concentration was adjusted to 5 μg for reverse transcription to cDNA using EasyScript^®^ First-Strand cDNA Synthesis SuperMix (TransGen, Beijing, China). All PCR amplification reactions were conducted in a volume of 20 μL, comprising 15 μL RNA, 4 μL of 5 All-in-One Reaction Mix for qPCR, and 1 μL of TransScript^®^ Uni All-in-One Enzyme Mix. PCR conditions were as follows: 50 °C incubation for 5 min. Subsequently, the cDNA was stored in a −80 °C refrigerator.

#### 2.5.3. Expression of the Immune-Related Genes

The transcriptional levels of immune-related genes interferon-gamma (IFN-γ) and Interleukin-10 (IL-10) were evaluated by quantitative RT-PCR assays with 18S rRNA (whose primer efficiency was verified to be 100%) as the reference (housekeeping) gene. The real-time quantitative PCR using SYBR Green Premix Pro Taq HS Qpcr Kit (ROX Plus) (AG, Guangzhou, China) was carried out in an ABI 7500 Real-time Detection System (Applied Biosystems, Waltham, MA, USA). All PCR amplification reactions were performed in a volume of 20 μL, containing 2 μL cDNA, 0.4 μL of each primer (10 μM), and 2 × Mix 10 μL. PCR conditions were as follows: 1 min at 95 °C for initial denaturation and 5 s at 95 °C and 30 s at 60 °C for 40 cycles. The expression level of the immune-related gene was determined using the formula F = 2^−∆∆Ct^, where ∆∆Ct = [(Ct, _target gene_ − Ct, _reference gene_) experimental] − [(Ct, _target gene_ − Ct, _reference gene_) control]. The primers used in this paper have been listed in Table 1.

### 2.6. Antioxidant Capacity Assessment

Liver tissue samples from Section 2.4 were used for antioxidant capacity assessment. Briefly, 0.05 g of tissue was accurately weighed and homogenized in nine volumes of normal saline to prepare a 10% (*w*/*v*) homogenate. The homogenate was centrifuged at 2500 rpm for 10 min, and the resulting supernatant was collected for subsequent analysis. The activities of alkaline phosphatase (AKP) and micro-reduced glutathione (GSH) were measured using commercial assay kits (Nanjing Jiancheng Bioengineering Institute, Nanjing, China). Total protein concentration was determined using the BeyoBCA Rapid Protein Assay Kit to normalize the enzymatic activities.

### 2.7. Statistical Analysis

All data in this paper were analyzed by GraphPad Prism 8.0 (GraphPad Software, Inc., San Diego, CA, USA). Each sample was assayed in triplicate, and results are reported as mean ± standard error of mean (SEM). Statistical differences between experimental and control groups were determined by two-way ANOVA and * at *p* < 0.05 and ** at *p* < 0.01.

## 3. Results

### 3.1. Breeding of Candidate Populations Resistant to LMBV

**Figure 1:**
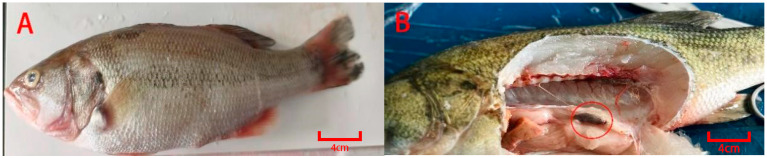
Symptoms of LMBV infection in largemouth bass. Body surface ulceration, fin bleeding, and the visceral signs, particularly splenomegaly, are evident. (**A**) fish fin ray hemorrhage; (**B**) fish splenomegaly. The red circles in the figure indicate that splenomegaly occurs in fish after infection with LMBV.

**Figure 2:**
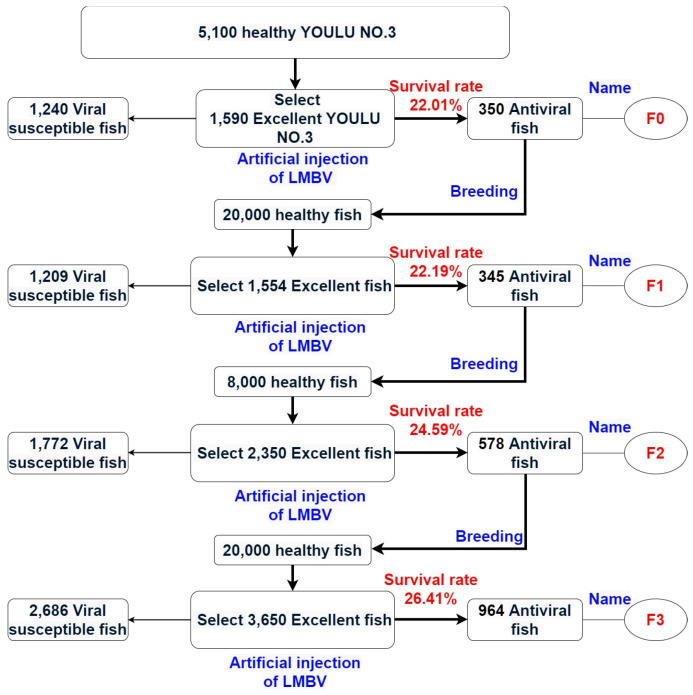
The selective breeding process of generations of Largemouth Bass resistant to LMBV. The survival rates for F0, F1, F2, and F3 were 22.01%, 22.19%, 24.59%, and 26.41%, respectively. The blue font indicates the specific operating procedures, and the red font represents the survival rate of each generation.

Subsequent to the virus challenge experiment (Figure 1), all test individuals across generations maintained a male-to-female ratio of 1:1. Specifically, Youlu No. 3 is a largemouth bass variety with a 70% market share in China, 1500 individuals of this variety were used as the base population, with 350 survivors (F0). For the F1 generation (bred from these surviving F0 individuals), 1554 individuals were subjected to the challenge test, resulting in 345 survivors; the F2 generation (bred from surviving F1 individuals) consisted of 2350 test individuals, with 578 survivors; and the F3 generation (bred from surviving F2 individuals) included 3650 test individuals, with 964 survivors (Figure 2). The corresponding survival rates were 22.01% (F0), 22.19% (F1), 24.59% (F2), and 26.41% (F3), respectively. A significant difference in survival rates was observed among the three selected generations, with F2 and F3 showing substantially higher survival rates than F1.

**Figure 3:**
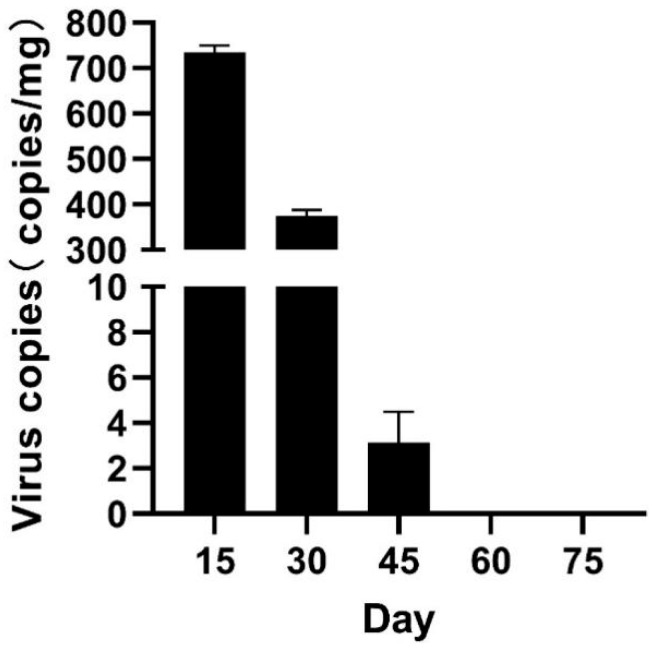
Residual detection of LMBV in candidate population post virus infection. In the study on disease-resistant fish screening, viral replication was detected every 15 days. The viral copy number consistently diminished from 15 days to 60 days post-challenge screening, ultimately reaching an undetectable level at 60 days.

The findings indicated that the copy number was 733 copies/mg at 15 days, 375 copies/mg at 30 days, 3.0 copies/mg at 45 days, and no detectable virus copy numbers at 60 days, demonstrating a consistent decline overall (Figure 3).

### 3.2. Comparative Experiment on Viral Resistance

**Figure 4:**
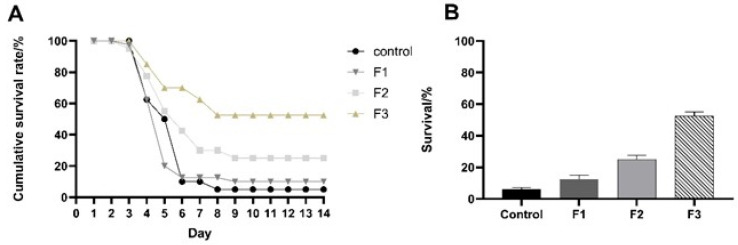
Cumulative survival rates (**A**) and survival rates (**B**) of largemouth bass infected with LMBV. All four groups begin to perish in significant quantities from the third day onward. By the seventh day, merely 10% of the control group persisted, 12.5% of F1 persisted, 30% of F2 persisted, and 62.5% of F3 persisted. On the ninth day, no additional fatalities transpired in the four groups, and by the fourteenth day, the survival rate reached a state of equilibrium. The ultimate survival rates for the control group were 5%, whereas those for F1, F2, and F3 were 10%, 25%, and 52.5%, respectively, surpassing the control group’s rate.

LMBV infection experiments were performed on F1, F2, and F3 generations to assess the efficiency of disease resistance and determine if there was an improvement in resistance. The cumulative survival rate results indicated that the control group, F1, F2, and F3, experienced significant mortality on the third day, with a deceleration in deaths observed by the seventh day. On the seventh day, survival rates were as follows: 10% for the control group, 12.5% for F1, 30% for F2, and 62.5% for F3. By the ninth day, no fatalities were recorded, resulting in ultimate survival rates of 5% for the control group, 10% for F1, 25% for F2, and 52.5% for F3 (Figure 4).

**Figure 5:**
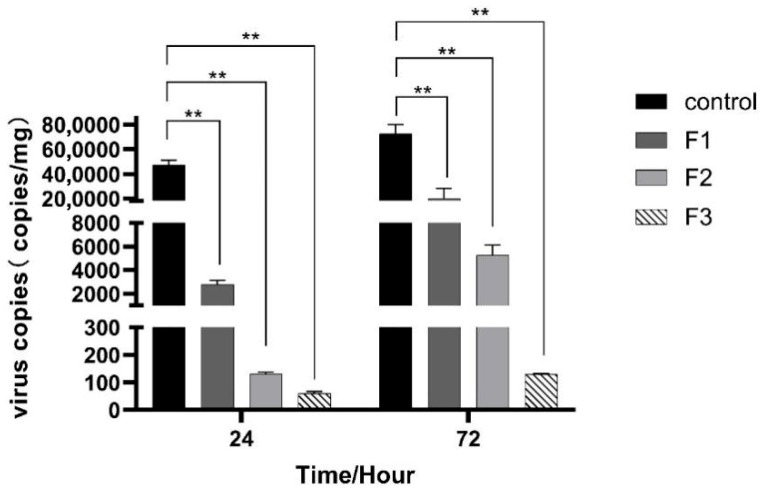
Virus copies in the spleen of largemouth bass infected with LMBV. At 24 hpi, the viral copy number in the control group was 472,564.0 copies/mg, which was markedly higher than that in F1 (2778.0 copies/mg), F2 (130.0 copies/mg), and F3 (59.5 copies/mg). At 72 hpi, the control group still maintained a high viral load (724,333.0 copies/mg), while F1 decreased to 201,170.0 copies/mg, F2 rose to 5259.0 copies/mg, and F3 only reached 129.0 copies/mg. Overall, the viral copy number in the control group significantly exceeded that of F1, F2, and F3, with a decrease in viral copy number corresponding to an increase in disease resistance formation (F3 < F2 < F1 < control at both time points). ** at *p* < 0.01.

Through the two designated time points, the viral copy number in the control group was markedly elevated compared to that in F1, F2, and F3 (Figure 5). At 24 hpi, the control group exhibited a copy number of 472,564.0 copies/mg, F1 recorded 2778.0 copies/mg, F2 had 130.0 copies/mg, and F3 measured merely 59.5 copies/mg. At 72 hpi, the control increased to 724,333.0 copies/mg, F1 decreased to 201,170.0 copies/mg, F2 rose to 5259.0 copies/mg, and F3 only reached 129.0 copies/mg. As disease resistance generation increased, the metabolic capacity of the virus became increasingly robust.

The survival study showed that F1, F2, and F3 exhibited a considerable improvement in resistance performance compared to the control group, thereby confirming the effectiveness and utility of disease resistance selection breeding in the genetic selection of complex traits.

### 3.3. Immune-Related Gene Expression Analysis

**Figure 6:**
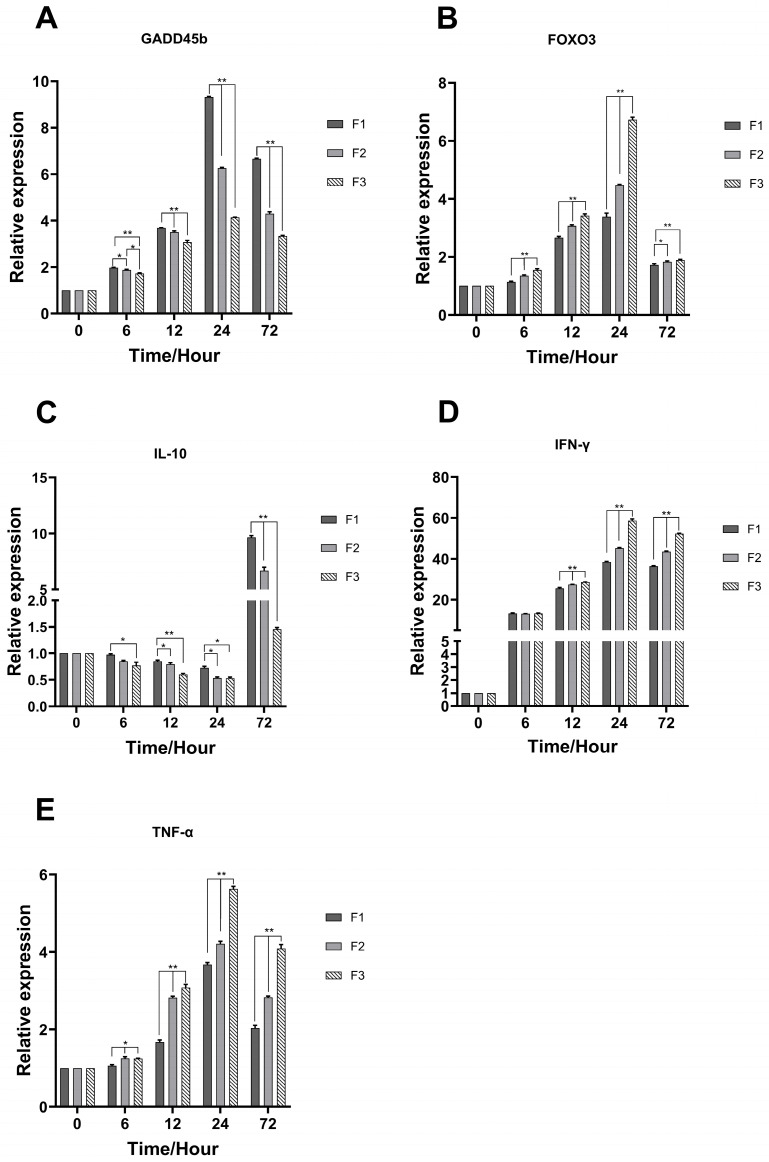
Relative expression of immune factors in the spleen of largemouth bass infected with LMBV. Total RNAs were extracted from the spleen tissues from F1, F2, and F3 at 6 h, 12 h, 24 h, and 72 h for detection of GADD45b (**A**), FOXO3 (**B**), IL-10 (**C**), IFN-γ (**D**), and TNF-α (**E**) post-infection using qRT-PCR. * At *p* < 0.05 and ** at *p* < 0.01.

In the spleen of F3, the expression levels of GADD45b, FOXO3, IL-10, IFN-γ, and TNF-α were significantly elevated (Figure 6). The results demonstrated that GADD45b peaked at 24 h, with increases of 2.25-fold and 1.50-fold relative to F1 and F2, respectively, before decreasing again at 72 h. FOXO3 exhibited up-regulation at 6 h, reaching a peak at 24 h, with increases of 2.03 and 1.47 times relative to F1 and F2, and followed by a temporary down-regulation at 72 h. IL-10 showed a declining tendency at 6 h and reaching its lowest level at 24 h, ultimately returning to its maximum level at 72 h, measuring 6.65 and 4.60 times more than the levels recorded in F1 and F2, respectively. IFN-γ demonstrated a consistent increase at 6 h, reaching a peak at 24 h, measuring 1.57 and 1.29 times higher than F1 and F2, respectively, followed by a rapid decrease at 72 h. However, TNF-α indicated a slight upregulation at 6 h, reaching a peak at 24 h, measuring 1.51 and 1.33 times greater than F1 and F2, respectively, followed by a rapid decrease to a low level at 72 h. The immune genes in F3 were upregulated compared to those in F1 and F2, indicating that F3 exhibited enhanced disease resistance relative to F1 and F2.

### 3.4. Antioxidant Capacity Assessment

**Figure 7:**
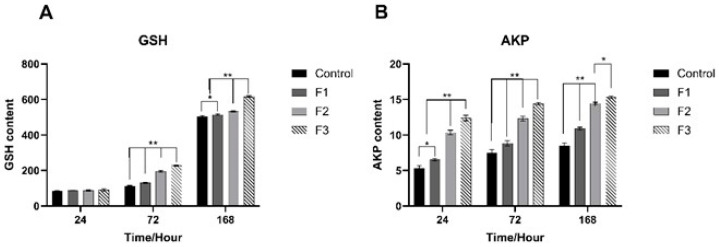
Content of GHS (**A**) and AKP (**B**) in the spleen of largemouth bass infected with LMBV. Over time, all three groups showed a gradual increasing trend, with the increasing trend of F3 being slightly higher than that of F2 and the control group. * At *p* < 0.05 and ** at *p* < 0.01.

Figure 7A illustrated that GSH levels exhibited an upward trend across the three designated time points in the four groups. At 24 h, the differences among the groups were not statistically significant. At 72 h, the control group measured 111 U/mg protein, F1 recorded 131, F2 reached 195, and F3 attained 227. At 168 h, the control group was at 505, F1 at 515, F2 at 532, and F3 at 617. Figure 7B illustrated that the AKP levels throughout the four groups exhibited an increasing trend through the three designated time points. At 24 h, the control group exhibited 5.3 U/mg protein, F1 displayed 5.4, F2 recorded 10.3, and F3 measured 12.3. At 72 h, the control group measured 7.3, F1 measured 8.8, F2 measured 12.2, and F3 measured 14.3. At 168 h, the control group measured 8.3, F1 measured 10.8, F2 measured 14.4, and F3 measured 15.3. The findings of GSH and AKP indicated that elevated levels of GSH and AKP correlate with enhanced antioxidant ability and increased disease resistance.

## 4. Discussion

Breeding for disease-resistant fish is becoming increasingly important due to heavier global reliance on aquaculture [21]. Thanks to the rapid development in DNA sequencing and fish genomics, new genomics-assisted breeding approaches have been developed and have greatly contributed to the improvement of fish disease resistance in this genomics era. Several fish breeding advancements using these technologies have been developed in aquaculture. Fuji et al. [22] employed marker-assisted selection technology to enhance *Paralichthys olivaceus* resistance to lymphocystis disease, elevating the survival rate of disease-resistant populations from 50% to 70%, demonstrating the efficacy of markers. Wasana et al. [23] propagated Nile tilapia resistant to *Streptococcus agalactiae* using genomic estimated breeding, increasing its survival rate by 21%. Xiao et al. [24] employ triploid breeding technology to breed triploid hybrids by interspecies crossing of female diploid red crucian carp with male allotetraploid, enhancing their market presence in China due to superior traits such as rapid growth, palatable flavor, robust disease resistance, and resilience to stress. Verrier et al. [25] utilized quantitative trait locus analysis to examine rainbow trout exhibiting resistance to rhabdovirus, revealing that the expression levels of antiviral genes in the resistant group were markedly elevated compared to those in the susceptible population, thereby confirming the functional relationship of QTLs. Zhao et al. [26] conducted a genome-wide association study to elucidate the genetic architecture of resistance to the parasite Cryptocaryon irritans in giant yellow croaker (*Larimichthys crocea*), thereby offering technical support for breeding for disease resistance, etc. In this study, the disease-resistant progeny was evaluated by LMBV infection tests, and the disease-resistant genes were identified and validated using GWAS technology. Therefore, the disease-resistant largemouth bass was developed by selective breeding and the purification of disease-resistant genes.

A significant difference in the survival rate among the three selective generations was observed, which illustrated that the three generations have different resistance abilities to the LMBV and that the selective breeding in the common largemouth bass to the virus had different validities. The survival rates of F2 and F3 were significantly higher than those of F1 and Control. Due to the survival rates of F2 and F3 showing differences, the consistently increased virus resistance across generations implied that the LMBV resistance ability can be inherited and that it can be enhanced by selective breeding. In summary, the F2 and F3 of largemouth bass exhibit significantly enhanced disease resistance compared to the F1 and control, with multiple lines of evidence supporting this conclusion.

IL-10, a key anti-inflammatory cytokine, plays a crucial role in modulating the immune response. It is primarily produced by a variety of myeloid cells and lymphoid cells [27,28]. Moreover, IL-10 performs a wide range of functions that reduce tissue damage caused by inflammation or infection [29]. TNF-α played an important role in the immunity and inflammation [30]. Collectively, IL-10 and TNF-α form a critical immune regulatory pathway against bacterial infections. Their upregulation by SA confirms their core value as disease resistance-related markers in LMB [31]. In our previous study [17], through genome-wide association analysis (GWAS), GADD45b was identified as a candidate gene associated with resistance to LMBV in largemouth bass. FOXO3 is a transcription factor of GADD45b and negatively regulates the expression of the gene. For disease resistance, the expression of GADD45b exhibited a specific temporal pattern within 72 hpi: it gradually increased from 0 to 24 hpi and then gradually decreased from 24 to 72 hpi. Importantly, F3, with relatively low expression levels of GADD45b compared to other groups, showed higher disease resistance. From immune-related gene expression, F3 shows lower expression levels of GADD45b and IL-10 and higher expression levels of FOXO3, IFN-γ, and TNF-α than F1, F2, and the control after LMBV infection. Therefore, F3 could activate -the Th1- and Th2-type immunity better than F1, F2, and the control.

The viral load in largemouth bass tissues can directly reflect the severity of disease caused by LMBV, making viral load analysis have potential predictive value [32]. Research data show that individual immunity has a statistically significant negative correlation with viral load. For example, vaccination with PRV particles can alleviate viral load in tissues by boosting immunity in fish [33]. Viral copy number exhibits a direct correlation with disease resistance, as evidenced by studies on fish showing that resistant strains consistently harbor lower viral copy numbers than susceptible counterparts during viral infections [34]. In our study, F3 has a markedly lower viral load in tissues compared to F1, F2, and the control, demonstrating a stronger ability to inhibit viral replication and clear pathogens.

AKP is involved in a series of physiological metabolic activities. AKP is a multifunction enzyme involved in a number of essential functions in living organisms [35]. GSH is the most prevalent non-protein thiol in animal cells. Its de novo and salvage synthesis serves to maintain a reduced cellular environment. GSH is the most powerful intracellular antioxidant and plays a role in the detoxification of a variety of electrophilic compounds and peroxides via catalysis by glutathione-S-transferases (GST) and glutathione peroxidases (GPx) [36]. Serum alkaline phosphatase (AKP) and glutathione (GSH) are key mediators of disease resistance in largemouth bass, with AKP enhancing innate immune function and GSH reinforcing antioxidant defense against Aeromonas dhakensis infection. Their significant dose-dependent elevation in sanguinarine-supplemented groups was tightly linked to reduced hepatic bacterial load and increased survival rates, confirming that synergistic improvements in immune enzyme activity and antioxidant capacity contribute to enhanced disease resistance [31]. Therefore, immune enzyme activities further support this advantage: F3 exhibits higher tissue activities of GSH and AKP than F1, F2, and the control, enhancing its ability to scavenge reactive oxygen species and resist pathogen invasion.

Notably, no significant differences in growth performance (body weight gain rate, specific growth rate) are observed between F1, F2, and F3, ensuring that the selection for disease resistance in F3 does not compromise its production traits. Therefore, the F3 generation of largemouth bass is a promising candidate for disease-resistant breeding, providing a valuable genetic resource to improve the sustainability of largemouth bass aquaculture.

## 5. Conclusions

This study effectively established an LMBV-resistant largemouth bass line, with the F3 generation appearing as the most promising candidate for disease-resistant breeding. Notably, the F3 progeny exhibited a significantly higher survival rate following LMBV infection compared to the F1, F2, and control groups—an enhancement closely linked to a favorable immune-related gene expression profile (downregulated GADD45b and IL-10, upregulated FOXO3, IFN-γ, and TNF-α) that confers robust antiviral immunity. Complementing this immune advantage, the F3 generation showed reduced viral load in tissues and elevated activities of antioxidant markers (GSH and AKP), while maintaining growth performance comparable to previous generations, with no compromise in weight gain or specific growth rate.

Importantly, the coupling of robust LMBV resistance and exceptional production features in the F3 generation addresses a fundamental bottleneck in largemouth bass aquaculture—balancing disease resilience with commercial viability. This discovery not only provides a rich genetic resource for sustainable aquaculture but also creates a platform for expanding disease-resistant breeding tactics in freshwater fish. By demonstrating the viability of iterative challenge-based selective breeding for LMBV resistance, our findings give practical guidelines for minimizing viral disease outbreaks and promoting the long-term stability of the largemouth bass farming industry. Concurrent with this, we are currently exploring multi-trait breeding approaches, such as integrating growth and reproductive traits to further elevate the strain’s comprehensive agricultural value.

## Figures and Tables

**Table 1:** Real-time PCR primers of immune factors.

Name	Sequences (5′-3)	Accession Numbers	Size
GADD45b-F	CTTTCTGCTGCGACAACGAC	XM_038699817.1	138 bp
GADD45b-R	GAGGGTTCGTGACCAGGATG		
FOXO3-F	ACAAGTACACCAAGTCCGCC	XM_038727152.1	196 bp
FOXO3-R	CTGGCGTTGGAATTAGTGCG		
IL-10-F	CTAGACCAGAGCGTCGAGGA	XM_038696252.1	103 bp
IL-10-R	CCAAGGCTGTTGGCAGAATC		
TNF-α-F	ATCTGCTGTGAATGCCGTGA	OK493793.1	203 bp
TNF-α-R	CGTCAGCCTGGATAGACGAC		
IFN-γ-F	TGCAGGCTCTCAAACACATC	XM_038707474.1	105 bp
IFN-γ-R	TGTTTTCGGTCAGTGTGCTC		
18s-F	TGACGGAAGGGCACCACCAG	XR_005440960.1	130 bp
18s-R	GCACCACCACCCACAGAATCG		

## Data Availability

The original contributions presented in this study are included in thearticle. Further inguiries can be directed to the corresponding authors.
